# Environmental parameters factors exploration on lettuce seed germination with hydrogel

**DOI:** 10.3389/fpls.2024.1308553

**Published:** 2024-03-07

**Authors:** Yanhua Huang, Yanbin Chang, Zheng Ni, Lizhi Wang

**Affiliations:** ^1^ Department of Industrial and Manufacturing Systems Engineering, Iowa State University, Ames, IA, United States; ^2^ School of Industrial Engineering and Management, Oklahoma State University, Stillwater, OK, United States

**Keywords:** hydroponics, lettuce, hydrogel, Hoagland, germination, timing, operations

## Abstract

Lettuce (*Lactuca sativa*) germination is sensitive to environmental conditions. Recently, hydrogel has received increased attention as an alternative media to soil for seed germination. Compared to soil seeding, hydrogel-aided germination provides more controlled seeding environments. However, there are still challenges preventing hydrogel-aided seed germination from being widely used in industry production or academic studies, such as hydrogel formulation variations, seeding operation standardization, and germination evaluation. In this study, we tested how the combination of multiple environmental conditions affect lettuce seed germination time, which is measured as the time needed for the first pair of leaves to appear (leaf emergence) or, alternatively, the third leaf to appear (leaf development). We found that germination time and success rate of two lettuce varieties (Iceberg A and Butter Crunch) showed different sensitivities to pH, Hoagland formulations and concentrations, light intensity, and hydrogel content. We have conducted statistical analysis on the correlation between germination time and these environmental conditions.

## 1 Introduction

Hydroponics applications have drawn significant attention recently based on several benefits, including water efficiency (Corrado et al., 2021), nutrient control (Yang and Kim, 2020), space optimization (Peiro et al., 2020), continuous production (Magwaza et al., 2020), improved crop quality (Shrivastava et al., 2023), and local food production (Gentry, 2019). Compared with traditional agriculture, hydroponic-based cultivation recycled the nutrient liquid to re-water the crop. A study showed a 64% reduction in water usage in hydroponically cultivated lettuce compared to soil-based cultivation (Majid et al., 2021). Without soil, the nutrients only come from circulated water. The nutrient conditions can be completely controlled by tailoring nutrients to specific plant requirements (Velazquez-Gonzalez et al., 2022). Automated water circulation systems are implemented in most of the hydroponic systems recently reported by the literature (Saad et al., 2021). Nutrient compositions, pH, temperatures, and watering timing within the water circulation system can be monitored and controlled via sensors and switches (Sihombing et al., 2019). Since most of the hydroponic systems are deployed indoors to enable complete environmental control, it also possesses the advantage of optimized space usage, continuous production, and enabling local food production. Commercial-grade hydroponic systems are profitable to ensure the viability of the business. Space optimization and continuous production are two common approaches to improve profitability (Tetreault et al., 2023). It also reduces the system’s dependency on the local climate. With air conditioning, atmosphere modification (CO_2_ and O_2_), and lighting control, hydroponic systems can produce suitable environmental conditions for crops regardless of the geological location (Zhang et al., 2022), therefore enabling local food production, reducing the duration between farms and dining tables.

Lettuce (*Lactuca sativa*) is attractive as a hydroponic crop in both industry production and academic research due to its fast growth (Lee et al., 2021), high yield (El-Nakhel et al., 2020), high water content (Majid et al., 2021), simple nutrient requirement (Sapkota et al., 2019), and lower temperature requirement (Gentry, 2019). For example, most of the lettuce varieties matured within 40 days (Zabel et al., 2020), with an average fresh weight of 150 g (Ezziddine and Liltved, 2021) with no less than 93% water content (Zabel et al., 2020). The simple nutrient requirement of hydroponically growing lettuce also reduces the production cost, enlarging the profit margins (Hosseini et al., 2021). With lower temperature preference, grown lettuce uses less heat to maintain the environmental temperature. Thus, it also reduces energy costs in cold locations like Sweden (Gentry, 2019). With these advantages, lettuce is one of the most popular crops grown hydroponically.

Germination time estimation is important for both industry production and academic research. It provides important time prediction to facilitate planning and scheduling, allocating resources, minimizing risks, simulating crop growth, predicting yield, and seed breeding. In the controlled environment agriculture industry, thin profit margins require tight operation scheduling and low-risk tolerance. Accurate germination estimation is helpful for the logistics, including transplanting, irrigation, modifying nutrients, and pre-setting lighting conditions. Germination has been studied on rice looking for cold tolerance genetic loci in detail (Thapa et al., 2020). Barley germination was also studied to identify loci associated with drought tolerance (Moursi et al., 2020). These insights can furthermore improve predicting and simulating accuracies for breeding research and genomic selections (Amini et al., 2022; Moeinizade et al., 2022; Wang, 2022).

Compared to soil cultivation, hydrogel is capable of providing more controlled seeding environment. As a mixture of hydrophilic polymers and water, most hydrogels are capable of retaining large amount of water, which provides the essential elements for seeds to germinate. Hydrogel has been validated for seed germination (Huang et al., 2022). With full control of its formulation, it provides similar mechanical pressure to seed as the physical contact of soil (Gao et al., 2021); delivers water and nutrients consistently (Ma et al., 2019); retains water from evaporation (Oladosu et al., 2022); protects seeds from water, temperature, and salt stress (Fadiji et al., 2022); protects seeds from diseases like bacteria, pathogens, and fungi (Tang et al., 2021); and provides a non-toxic and bio-active environment (Fiorati et al., 2021). Despite these benefits, hydrogel-aided seed germination also faces several challenges. Compared with seed germination cubes such as gro-blocks (Barnett, 1986), hydrogel is much expensive. However, it also provides more benefits than gro-block, including temperature stress protection, nutrient customization, root phenotype visualization, and tunable mechanical gripping (An et al., 2022).

In this study, we conducted three experiments to explore the sensitivity of lettuce seed germination to environmental parameters, including hydrogel formulation, nutrient composition, pH, and light intensity during the germination process.

## 2 Materials and methods

### 2.1 Materials

Methylcellulose (MC) hydrogel was donated by J. Rettenmaier & Söhne (Schoolcraft, MI). All other chemicals were purchased from Thermo Fisher Scientific (Waltham, MA). Iceberg A lettuce (*Lactuca sativa* var. *crispa*, crisphead group) and Butter Crunch lettuce (*Lactuca sativa* var. *capitata*, butterhead group) were purchased from Burpee Garden Products Co (Warminster, PA). We used two versions of modified Hoagland solutions, formulated as listed in **Table 1** (MH1) and in **Table 2** (MH2) according to Brechner et al. (1996).

**Table 1:** Modified Hoagland solution 1 (MH1) formula for 100 L.

Chemicals	Mass (mg)
Sulfuric acid	0.1
Sodium phosphate tribasic dodecahydrate	37,704.8
Ammonium molybdate tetrahydrate	3.4
Iron(II) sulfate heptahydrate	507.2
Calcium hydroxide	38,924.1
Manganese(II) chloride	26.0
Iron(III) chloride	0.4
Potassium tetraborate tetrahydrate	78.1
Copper sulfate pentahydrate	5.5
Ammonium chloride	0.1
Ethylenediaminetetraacetic acid (EDTA)	19,454.0
Zinc nitrate hexahydrate	10.5
Potassium hydroxide	39,635.7
Manganese(II) chloride	22.5
Magnesium sulfate	24,043.3
Ammonium nitrate	60,120.2

**Table 2:** Modified Hoagland solution 2 (MH2) formula for 100 L, based on Brechner et al. (1996).

Chemicals	Mass (mg)
Calcium nitrate	47,404.92
Potassium nitrate	43,096.86
Ammonium nitrate	1,365.57
Sprint 330 iron—DTPA	913.63
Monobasic potassium phosphate	13,265.57
Potassium sulfate	1,064.82
Magnesium sulfate	11,997.54
Magnesium(II) chloride	41.62
Zinc sulfate	56.09
Boric Acid	90.71
Copper(II) sulfate pentahydrate	9.10
Ammonium molybdate	5.85

### 2.2 Experimental design

Three trials were designed to test the germination performance of two varieties of lettuce in different environmental conditions.

**Trial 1: Germination with MH1 solution**

In Trial 1, Iceberg A and Butter Crunch lettuce seeds were seeded in a 96-well plate with 200 µL conditioned MH1 to directly evaluate the different performances of the conditioned Hoagland solution. To facilitate the water absorption and swelling process during the germination initiation stage, reduced concentrations of MH1 were chosen to reduce the osmotic pressure over the seed’s skin, including one-third, one-fourth, and one-fifth of the original Hoagland solution concentration. To explore the impacts of acidity to the germination results, different diluted MH1’s pH values were used at 4, 5, 6, and 7 with 2% error. Deionized waters (DI) with different pH were also used to germinate seeds as the control group. For each condition, three seeds were placed in different wells as replicates. The germination process was executed under 25°C with par 30, 2,000 lumens, 5,000K LED lights at all times to stimulate germination. The motivation of using continuous light in Trial 1 is the discovery that seed germination rate of *Arabidopsis thaliana* responded positively to continuous red light (Millar et al., 2010), although it was in microgravity.

**Trial 2: Germination with MH1-formulated hydrogel**

Trial 2 aims to verify the compatibility between modified MH1 and MC hydrogel. Since the mechanical strength of hydrogels is directly related to the concentration of non-single valent cation salt, lower dry MC powder content (6% w/w) was mixed with the different concentrations of MH1 when formulating the hydrogel for seed germination substrate, compared to the previous minimum of 8% (Jiang et al., 2021). Seeding orientation variations were also varied, differentiated between vertical and horizontal scenarios. In the previous study, seeds with horizontal orientation swelled and sprouted superior to other orientations in terms of swelling ratio and sprout length (Huang et al., 2022). In this study, for each well of the six-well plate with lid, 0.6 g of MC powder were mixed with 1.4 mL of different conditioned Hoagland solutions used in Trial 1 to formulate 2 g of hydrogel. pH modification was conducted by adding hydrochloric acid and sodium hydroxide, since sulfur (S) in sulfuric acid and potassium (K) in potassium hydroxide are key nutrients in the MH1. In each well, 12 seeds were seeded vertically and four horizontally, 1 mm below the top surface of the cast hydrogel. There was a 24-h wait between hydrogel formulation and seeding due to the homogenization of the ions and migration and cross-linking of the hydrogel polymer chain. The germination process was executed under 21°C with mixed LED lights ranging from 4,000 K to 5,500,K at all times.

**Trial 3: Germination with MH2-formulated hydrogel**

In Trial 3, seed germination was carried out in the MC hydrogel with non-full factorial formula modification mapping based on MH2 for a maximum of 8 days. The formula mapping parameters and their conditions are listed in **Table 3**. Prior to adding MC powders, pH was measured. A total of 50 gels were cast in the ×100, 100-mm plastic Petri dish with a lid. After six Iceberg A lettuce seeds were seeded in each hydrogel, all 50 hydrogels were placed under two 24-W 5,000 K LED lights for 8 days without changing positions. The light photosynthetic photon flux density (PPFD) of each dish was logged for data analysis. We explored the following independent variable as experimental matrix:

- MH2: concentration times of original MH2 condition; unit, X;
- NH_4_NO_3_: ammonium nitrate beyond MH2 formulation; unit, ppm;
- KOH: addition of potassium hydroxide beyond MH2 formulation; unit, ppm;
- PO_4_: addition of phosphate ions beyond MH2 formulation; unit, ppm;
- Citric: addition of citric acid; unit, ppm;
- MC: weight percentage of MC polymer used in the hydrogel formulation; unit, %. w/w;
- pH: acidity/alkalinity of the formulation; unitless;
- PPFD: light photosynthetic photon flux density; unit *µ*mol*/*s*/*m^2^.

**Table 3:** A total of 50 non-full factorial formulas for methyl cellulose hydrogels based on modified Hoagland solution 2.

No.	MH2 (x)	NH_4_NO_3_ (ppm)	KOH (ppm)	KH_2_PO_4_ (ppm)	K_2_HPO_4_ (ppm)	Citric Acid (ppm)	MC (%, w/w)
1	0	0	10	0	5	20	6
2	0	0	30	0	5	10	8
3	0	0	30	5	20	50	10
4	0	5	10	5	20	20	6
5	0	5	20	10	80	10	8
6	0	5	30	0	5	50	10
7	0	10	0	0	5	10	10
8	0	10	0	5	20	50	8
9	0	10	20	5	20	20	10
10	0	35	0	10	80	10	6
11	0	35	10	10	80	20	8
12	0	35	20	10	80	50	6
13	0.5	0	0	5	20	20	8
14	0.5	0	10	5	20	10	10
15	0.5	0	20	0	5	20	8
16	0.5	0	30	10	80	10	6
17	0.5	5	0	5	20	50	8
18	0.5	5	0	10	80	50	10
19	0.5	5	20	0	5	20	10
20	0.5	10	10	0	5	50	6
21	0.5	10	30	0	5	10	8
22	0.5	10	30	10	80	20	6
23	0.5	35	10	10	80	50	10
24	0.5	35	20	0	5	10	10
25	0.5	35	30	5	20	10	6
26	1	0	0	5	20	10	6
27	1	0	0	10	80	20	10
28	1	0	20	0	5	50	10
29	1	5	10	0	5	10	8
30	1	5	10	10	80	10	8
31	1	5	20	5	20	50	6
32	1	10	10	5	20	10	10
33	1	10	20	10	80	50	6
34	1	10	30	10	80	20	8
35	1	35	0	0	5	20	6
36	1	35	30	0	5	20	10
37	1	35	30	5	20	50	8
38	1.5	0	10	10	80	50	10
39	1.5	0	20	10	80	10	8
40	1.5	0	30	0	5	50	6
41	1.5	5	0	0	5	10	6
42	1.5	5	10	0	5	20	6
43	1.5	5	30	5	20	20	10
44	1.5	5	30	10	80	10	10
45	1.5	10	0	10	80	20	10
46	1.5	10	10	0	5	50	8
47	1.5	10	20	5	20	10	6
48	1.5	35	0	0	5	50	8
49	1.5	35	10	5	20	10	10
50	1.5	35	20	5	20	20	8

### 2.3 Germination score

Based on previously used metrics for germination performance (Brechner et al., 1996; Egbuikwem et al., 2020), we propose a germination score as a performance metric, which requires the following definition of germination time (*D*) and success rate (*R*).

- Leaf emergence *D*_2_: average number of days needed for the first pair of leaves to appear, capped at day 8.
- Leaf development *D*_3_: average number of days needed for the third leaf to appear, capped at day 10.
- *D*_T_: average number of days between the first pair of leaves and the third leaf.
- Germination rate *R*_2_: percent of seeds that have reached leaf emergence by the eighth day of germination.
- Germination rate *R*_3_: percent of seeds that have reached leaf development by the 10th day of germination.
- Germination score *G*_2_ = (8 − *D*_2_) · *R*_2_.
- Germination score *G*_3_ = (10 − *D*_3_) · *R*_3_.

## 3 Results and discussions

### 3.1 Trial 1 results

**Figure 1:**
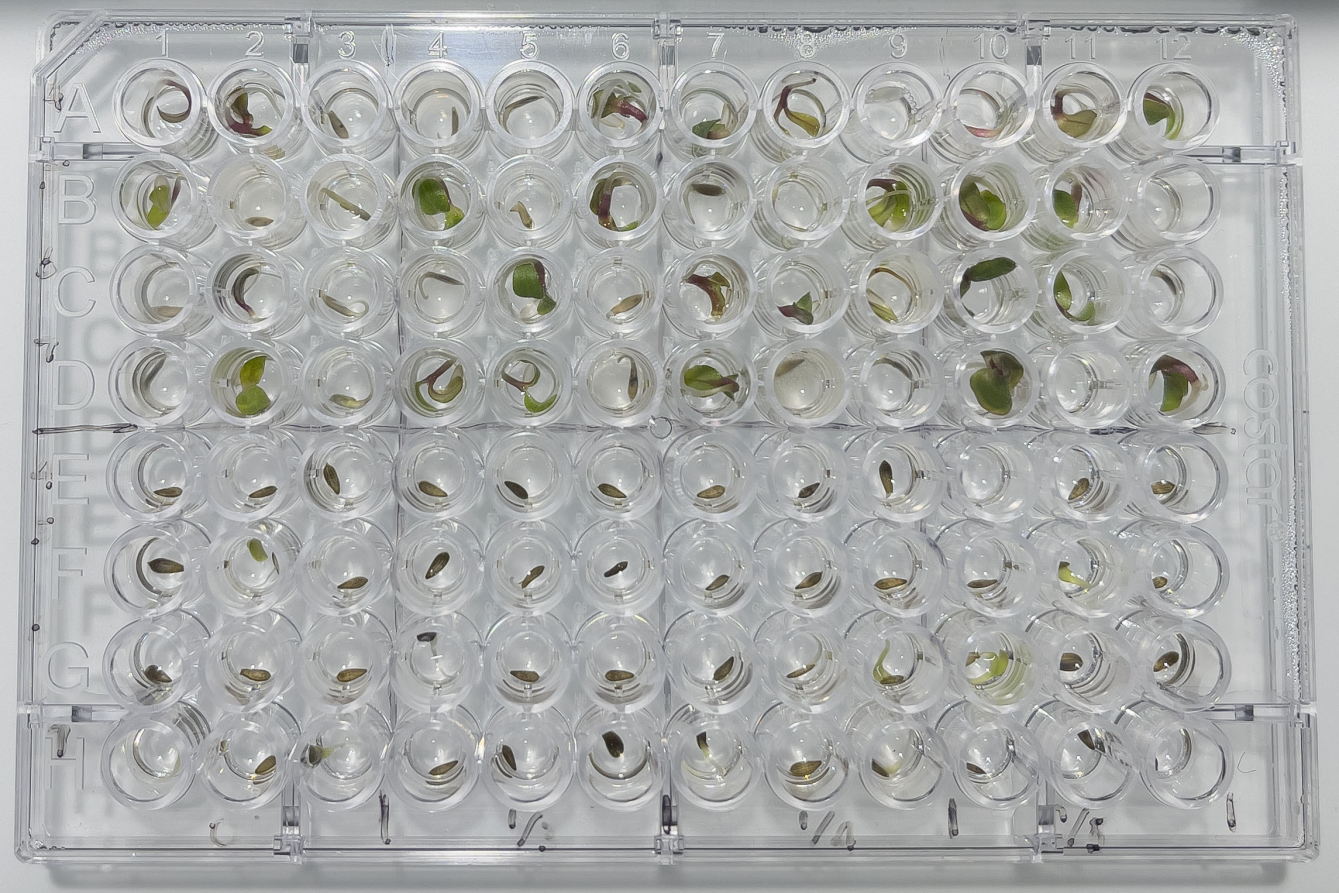
In Trial 1, day 4 of Iceberg A (top half) and day 3 of Butter Crunch (bottom half) lettuce germination without MC hydrogel in a 96-well plate.

**Figure 2:**
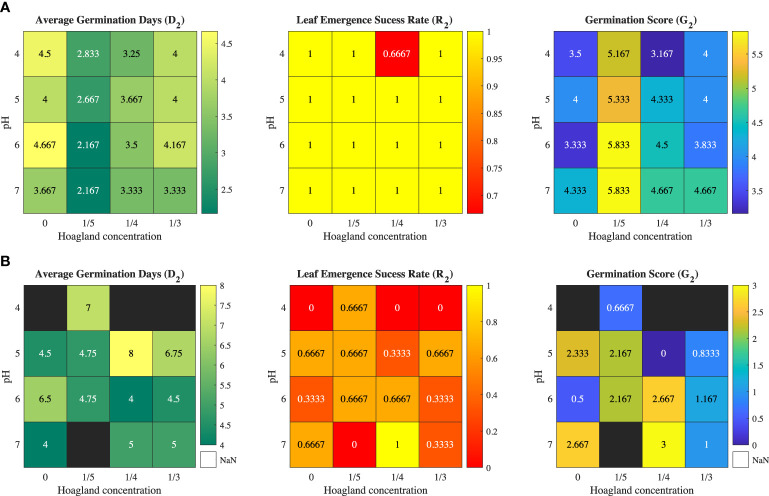
Trial 1: Heat maps of average germination time (D2), success rate (R2), and germination score (G2) for leaf emergence without methylcellulose hydrogel for **(A)** Iceberg A lettuce and **(B)** Butter Crunch lettuce.

The Iceberg A and Butter Crunch lettuce seeds in the 96-well plate are shown in **Figure 1**. The germination time *D*_2_, success rate *R*_2_, and germination score *G*_2_ for the two varieties are show in **Figures 2A, B**. The shortest average germination time *D*_2_ for Iceberg A was 2.167 days, which was under the condition of pH being 6 or 7 and MH1 concentration being one-fifth level of original conditions. Butter Crunch lettuce had a lower germination success rate and took a longer time to germinate. There were four cases that Butter Crunch lettuce could not germinate, inducing 0, 1/3, and 1/4 MH1 solution at pH = 4 and 1/5 MH1 at pH =7, which are indicated by the black blocks in **Figure 2B**.

Germination solution impacted the germination rate of the two varieties differently. Seeds require absorbing a significant amount of water before reactivating their bio-activity from dormancy. An overcharged nutrient solution may depress the swelling process, which delays or prohibits seeds from germination. On the other hand, changing pH can alter enzyme activities at the cell membrane. Unsuitable pH conditions could lead to calcium or magnesium ions disorder and delayed germination (Borhannuddin Bhuyan et al., 2019).

### 3.2 Trial 2 results

**Figure 3:**
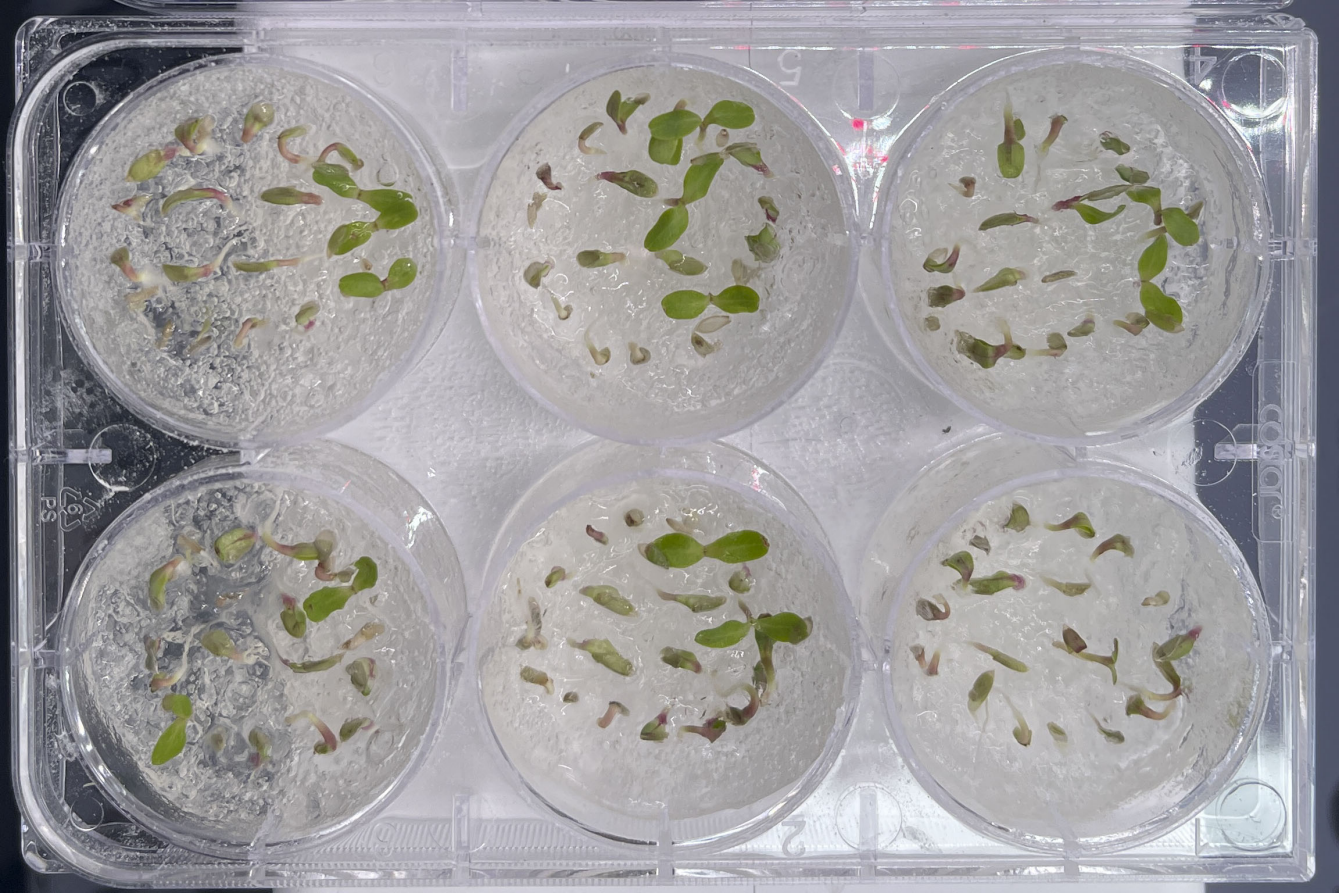
In Trial 2, day 3 of Iceberg A lettuce germination with methyl cellulose hydrogel formulated with modified Hoagland solution 1 in six-well plate. In each well, the center four seeds were seeded horizontally, and the rest were seeded vertically.

**Figure 4:**
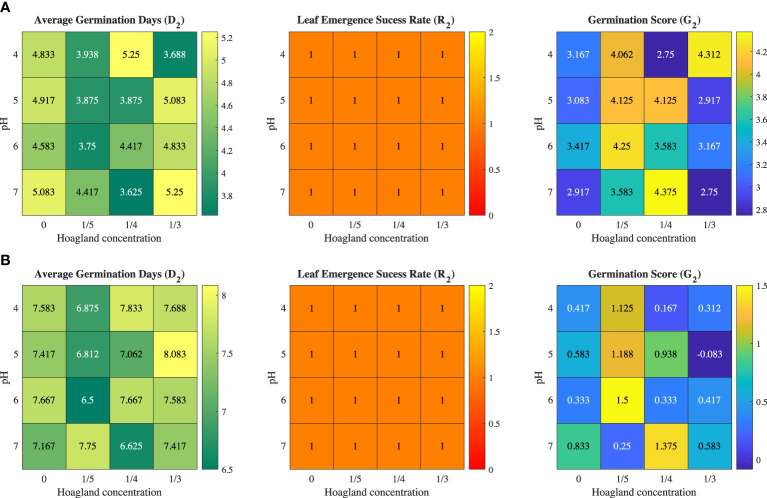
Trial 2: Heat maps of average germination time (D2), success rate (R2), and germination score (G2) leaf emergence with MC hydrogel for **(A)** Iceberg A lettuce and **(B)** Butter Crunch lettuce.

**Figure 3** shows the Iceberg A and Butter Crunch lettuce seeds in the six-well plate with MH1-formulated MC hydrogel. The germination time *D*_2_, success rate *R*_2_, and germination score *G*_2_ for the two varieties are show in **Figures 4A, B**. These figures showed that the shortest germination times *D*_2_ for Iceberg A and Butter Crunch were 3.625 days and 6.5 days, respectively, which were noticeably longer than the respective 2.167 days and 4 days from Trial 1. Germination score G_2_ also showed similar trends.

The presence of the MC hydrogel impacted seed germination significantly. When seeded in hydrogels, the seed experienced mechanical compression during germination. Due to the water retention from the hydrogel, less water was available for seeds to swell, therefore delaying the germination process (Bauli et al., 2021). However, hydrogel significantly improved germination success rate. Compared with 56% without hydrogel, germination with MH1-formulated hydrogel provide a 64% of success rate. However, the increase in average germination time offsets the improvement of the success rate role in the germination score.

When seeded in hydrogel, seed orientation significantly impacts the average germination time. Since the dimension of the lettuce seeds is relatively small compared to soybean, it is difficult to distinguish from hilum up or down without a desktop microscope or magnifying glass (Huang et al., 2022). It is safe to plant the seed sideways to ensure less discrepancy induced from seed orientation, as shown in **Figure 3**. Based on the results, we found that excess sodium and chlorine-rich MH1 formulation posed salt stress to germination.

### 3.3 Trial 3 results

In Trial 3, we used a different version of Hoagland formula, MH2, with reduced sodium (Na) and chlorine (Cl) in the nutrient solution. Although inexpensive, Na and Cl salts can significantly shift the osmotic pressure, which depresses swelling (Serrano and Gaxiola, 1994). The MH2 formula has only one ingredient, manganese (II) chloride (MnCl_2_), which contained Na or Cl. Since Mn is a micronutrient in the Hoagland formula, the amount of Cl is negligible. Six Iceberg A lettuce seeds were seeded together in a 10 × 10 mm.

**Figure 5:**
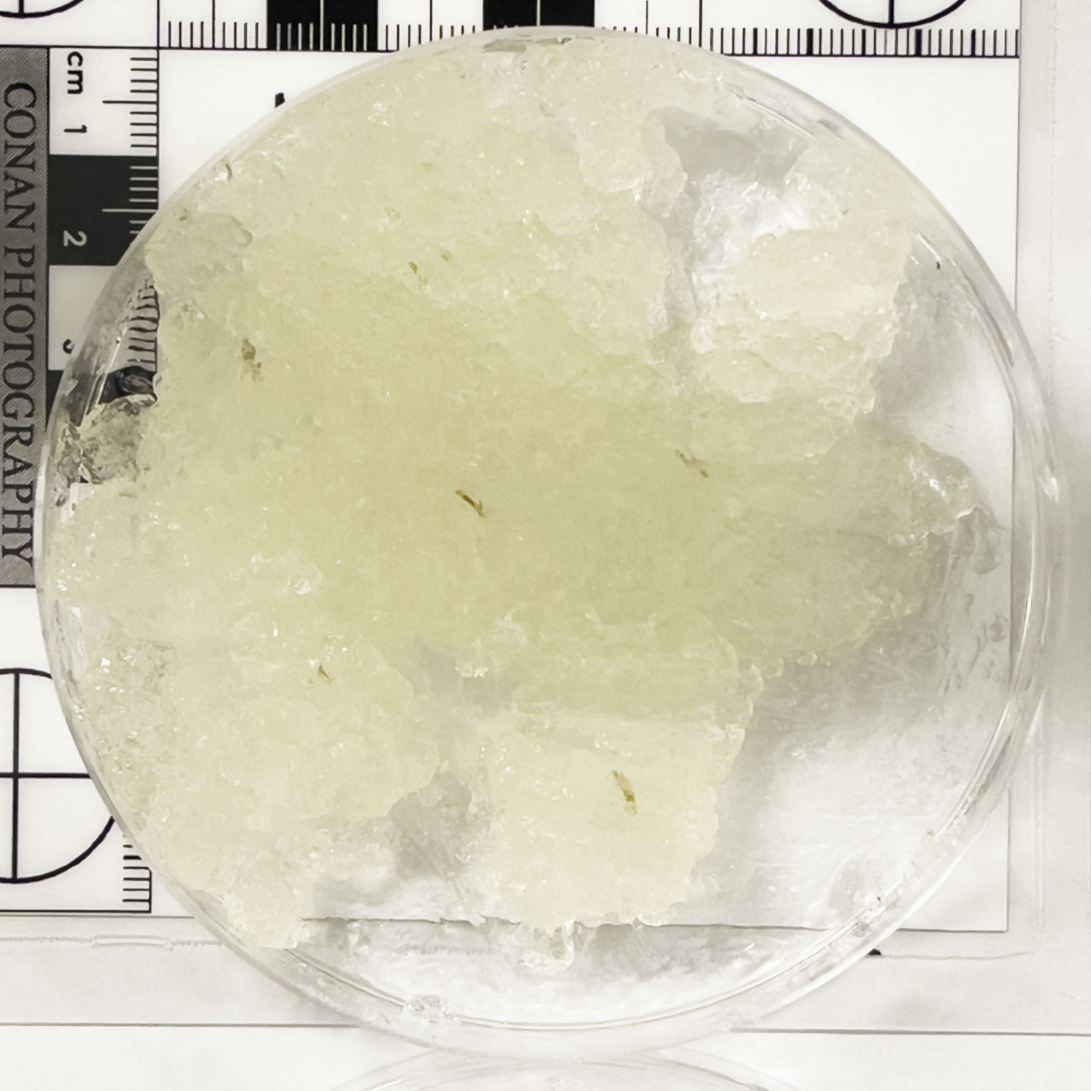
In Trial 3, day 0 of seeded Iceberg A lettuce in methyl cellulose hydrogel formulated with modified Hoagland solution 2 No. 9.

Petri dish with 30 mL MH2-formulated hydrogel, as shown in **Figure 5**. We only used Iceberg A seeds in Trial 3 because they failed to germinate in several cases in Trial 1 and had a much higher germination score than Butter Crunch in both Trials 1 and 2. Since the previous set showed that pH impacted the germination significantly, we altered the pH by adding different amounts of KOH and citric acid to avoid Na and Cl toxicity. Since nitrogen (N), phosphorus (P), and potassium (K) are the three macronutrients being studied extensively, minor N, P, and K modifications based on the MH2 formula were also introduced in the design of experiments.

More parameters were added in the experimental design to further explore the potential of hydrogel in the perspective of assisting lettuce seed germination. It was found (Millar et al., 2010) that seed responds to photonic energy significantly under microgravity conditions. Previous studies showed significant differences when seeds were sprouted in hydrogels with different polymer concentrations (Huang et al., 2022). In this study, we experimented with the same condition (6, 8, and 10% w/w hydrogel content).

Comparing MH1- with MH2-formulated hydrogels, average germination time (D_2_) were shortened from 4.46 days to 4.25 days, validating the chlorine toxicity delay. The average D_3_ was 6.28 days. In comparison, the standard industrial estimate is 7–10 days (Hamilton, 2018), and 10 days (Egbuikwem et al., 2020) and 11 (Brechner et al., 1996) days for pre-transplantation.

**Figure 6:**
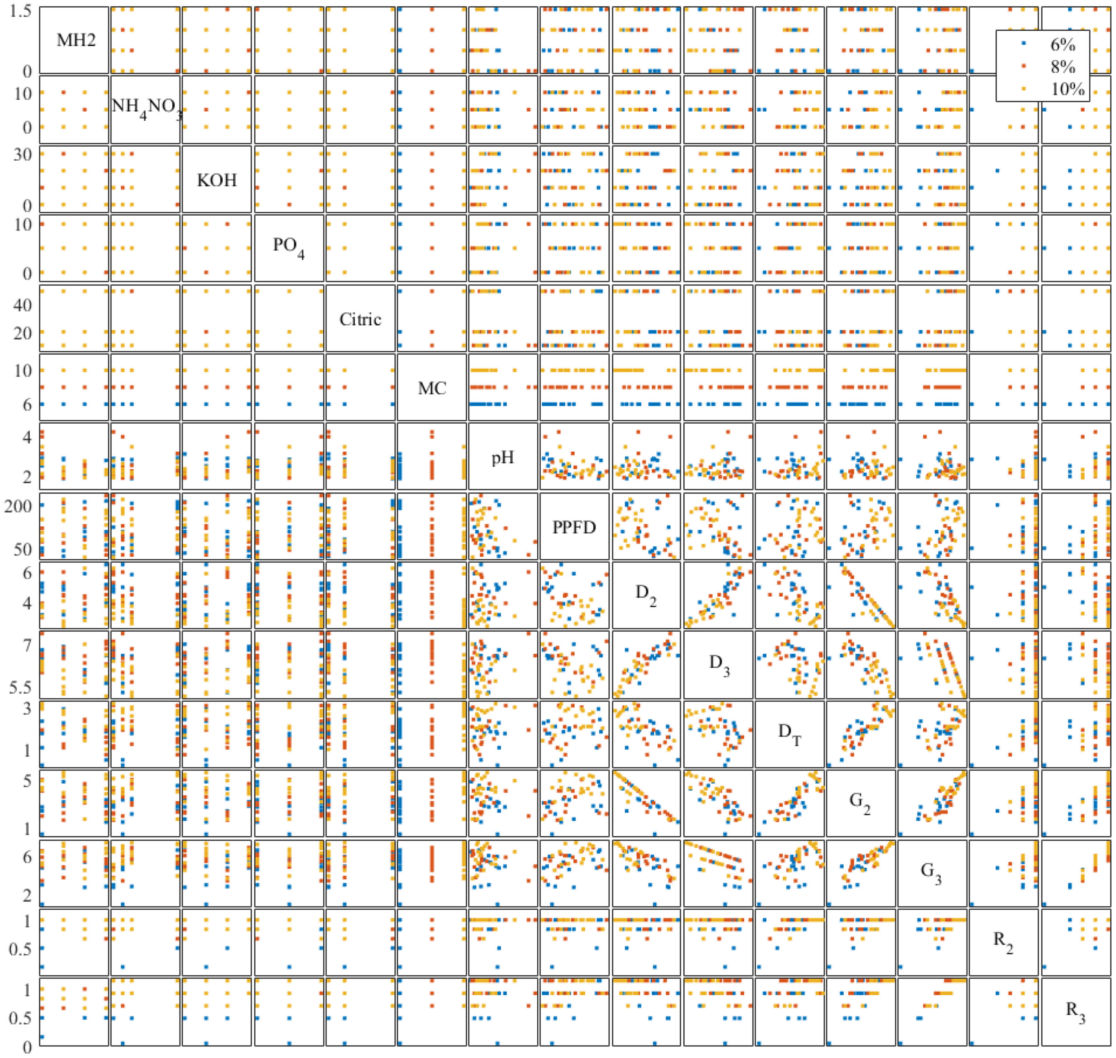
Trial 3: multivariate scatter plot (each dot represents the average of six replicate seeds’ condition germinated in one of the 50 gels as listed in **Table 3**) between controlled formulation variables and germination evaluators on the modified Hoagland 2-formulated methyl cellulose hydrogels. Controlled formulation variables and germination evaluators’ descriptions can be found in **Table 3** and *Section 2.3*, respectively.

**Figure 7:**
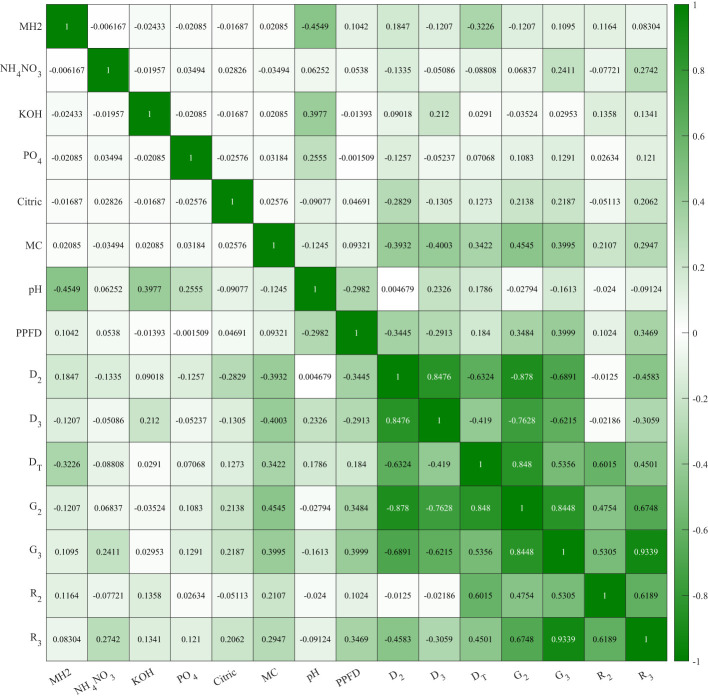
Trial 3: correlations between controlled formulation variables and germination evaluators on the modified Hoagland solution 2-formulated methyl cellulose hydrogels.

We analyzed the correlation between all controlling variables and evaluators to understand the effectiveness of independent variables on seed germination. Previous studies showed environmental factors impacting on lettuce germination including temperature (Hayashi et al., 2008), lighting conditions (Li et al., 2021), oxygen content (Yasin and Andreasen, 2016), and CO_2_ content (Luo, 2020). The multivariate scatter plot and correlation coefficients are shown in **Figures 6**, **7**. As grouped with MC content (%, w/w) in **Figure 6**, scatter plot D_2_ vs. D_3_, D_T_ vs. G_2_, and G_2_ vs. G_3_ showed clear color trends that indicate a high correlation between these evaluators and MC content. KOH and PO_4_ showed 0.3977 and 0.2555 correlations, respectively, with pH in **Figure 7**. The correlation between pH and KOH alternation is expected. As phosphate salts are acting as buffer, the addition of KOH increases the pH. Cellulose-based hydrogels change their mechanical integrity during the pH change, as demonstrated in Jiang et al. (2021). A −0.4549 correlation between pH and MH2 signifies the buffering capability of Hoagland solution. MH2 concentration had a −0.3226 correlation with D_T_. Both pH and MH2 correlations confirm that chlorine toxicity from MH2 significantly reduced the germination progress.

**Figure 8:**
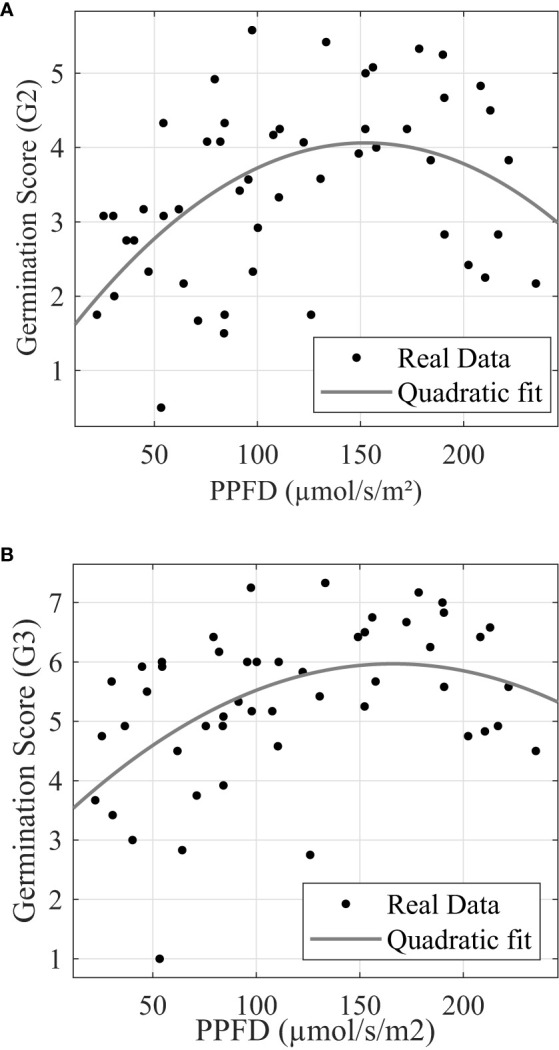
Trial 3: Leaf emergence **(A)** and leaf development **(B)** response to photonic energy presents stress. **(A)** G_2_ response to photosynthetic photon flux density. **(B)** G_3_ response to photosynthetic photon flux density.

Similar to post-germination plant modeling, light stress also depresses the seed germination process significantly after PPFD exceeds certain levels. **Figures 8A, B** show that G_2_ and G_3_ reached maximum at 152.30 µmol*/*s*/*m^2^ and 166.14 µmol*/*s*/*m^2^, respectively.

**Figure 9:**
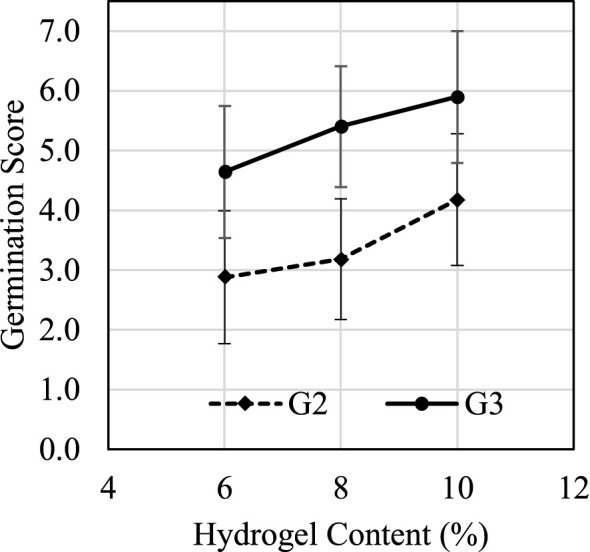
Trial 3: G2 and G3 response to hydrogel concentration.

**Figure 10:**
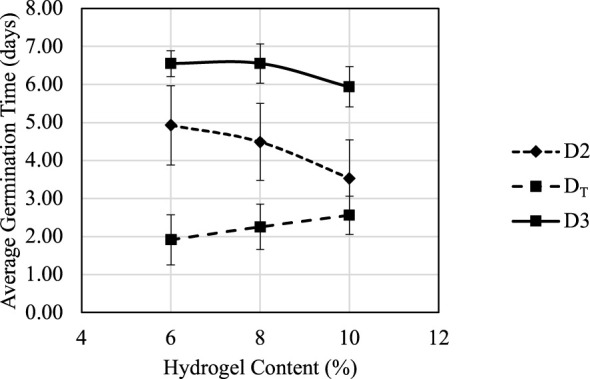
Trial 3: D2, D3, and D_T_ response to hydrogel concentration.

MC content plays an important role during the germination process. **Figure 9** suggested that germination score G_2_ had a positive correlation with hydrogel content between 6% and 10%. **Figure 10** shows how the average D_2_, D_3_, and D_T_ change under different hydrogel content between 6% and 10%. When hydrogel content increased, the porosity of fully swelled hydrogel also increased due to water content decrease; thus, additional oxygen could be trapped and defused in the hydrogel for roots’ immediate access. Increased porosity provides additional space for roots to expand, which also reduce germination times D_2_ and D_3_ and increase germination scores G_2_ and G_3_. The air and space increases are crucial during the swelling process for seeds’ respiration and volume span. Higher hydrogel content also led to an increase in D_T_ because it suppressed nutrient absorption and delayed the nutrient source transformation from seeding to cloning stages.

## 4 Conclusion

In this study, we experimentally explored the effects of multiple environmental parameters on lettuce seed germination. We found that pH, light intensity, cultivation substrate hardness, and porosity have the largest influences on lettuce seed germination with respect to germination time and success rate. In the meantime, MC hydrogel reduced the germination time by providing beneficial conditions for the seed to undergo soil-less germination, including the benefits of regulating water, nutrient supply to oxygen access, and carbon dioxide absorption, providing mechanical grip to protect against diseases.

This study is not without its limitations. For example, we conducted all experiments under the same temperature and humidity conditions without exploring their impact on germination. Salt stress from N, P, and K was also studied within a relatively narrow range. Future research should explore the effects of more environmental parameters over wider ranges on germination. Understanding such effects will not only provide more scientific insight on plant physiology but also commercial value for the controlled environment industry.

## Data availability statement

The original contributions presented in the study are included in the article/supplementary material. Further inquiries can be directed to the corresponding author.

## Author contributions

YH: Conceptualization, Data curation, Formal analysis, Investigation, Methodology, Validation, Visualization, Writing – original draft, Writing – review & editing. YC: Data curation, Methodology, Writing – review & editing, Formal analysis. ZN: Data curation, Methodology, Writing – review & editing, Formal analysis. LW: Funding acquisition, Project administration, Supervision, Writing – review & editing, Formal analysis, Methodology, Resources.

## References

[B1] Amini F. Hu G. Wang L. Wu R. (2022). The l-shaped selection algorithm for multitrait genomic selection. Genetics 221. doi: 10.1093/genetics/iyac069 PMC9252277 35482523

[B2] An B. Wang Y. Huang Y. Wang X. Liu Y. Xun D.. (2022). Engineered living materials for sustainability. Chem. Rev. 123, 2349–2419. doi: 10.1021/acs.chemrev.2c00512 36512650

[B3] Barnett J. P. (1986). Producing southern pine seedlings in containers (US Department of Agriculture, Forest Service, Southern Forest Experiment Station), 59.

[B4] Bauli C. R. Lima G. F. de Souza A. G. Ferreira R. R. Rosa D. S. (2021). Eco-friendly carboxymethyl cellulose hydrogels filled with nanocellulose or nanoclays for agriculture applications as soil conditioning and nutrient carrier and their impact on cucumber growing. Colloids Surf. A: Physicochem. Eng. Aspects 623, 126771. doi: 10.1016/j.colsurfa.2021.126771

[B5] Borhannuddin Bhuyan M. H. M. Hasanuzzaman M. Nahar K. Mahmud J. A. Parvin K. Bhuiyan T. F.. (2019). Plants behavior under soil acidity stress: insight into morphophysiological, biochemical, and molecular responses (Springer International Publishing), 35–82. doi: 10.1007/978-3-030-06118-02

[B6] Brechner M. Both A. Staff C. (1996). Hydroponic lettuce handbook. Cornell Controlled Environ. Agric. 834, 504–509.

[B7] Corrado G. Micco V. D. Lucini L. Miras-Moreno B. Senizza B. Zengin G.. (2021). Isosmotic macrocation variation modulates mineral efficiency, morpho-physiological traits, and functional properties in hydroponically grown lettuce varieties (lactuca sativa l.). Front. Plant Sci. 12. doi: 10.3389/fpls.2021.678799 PMC8212932 34149779

[B8] Egbuikwem P. N. Mierzwa J. C. Saroj D. P. (2020). Assessment of suspended growth biological process for treatment and reuse of mixed wastewater for irrigation of edible crops under hydroponic conditions. Agric. Water Manage. 231, 106034. doi: 10.1016/j.agwat.2020.106034

[B9] El-Nakhel C. Petropoulos S. A. Pannico A. Kyriacou M. C. Giordano M. Colla G.. (2020). The bioactive profile of lettuce produced in a closed soilless system as configured by combinatorial effects of genotype and macrocation supply composition. Food Chem. 309, 125713. doi: 10.1016/j.foodchem.2019.125713 31708345

[B10] Ezziddine M. Liltved H. (2021). Quality and yield of lettuce in an open-air rooftop hydroponic system. Agronomy 11, 2586. doi: 10.3390/agronomy11122586

[B11] Fadiji A. E. Santoyo G. Yadav A. N. Babalola O. O. (2022). Efforts towards overcoming drought stress in crops: Revisiting the mechanisms employed by plant growth-promoting bacteria. Front. Microbiol. 13. doi: 10.3389/fmicb.2022.962427 PMC9372271 35966701

[B12] Fiorati A. Linciano C. Galante C. Raucci M. G. Altomare L. (2021). Bioactive hydrogels: Design and characterization of cellulose-derived injectable composites. Materials 14, 4511. doi: 10.3390/ma14164511 34443033 PMC8398032

[B13] Gao Y. Zhao X. Han X. Wang P. Zheng W. J. (2021). Soft actuator based on metal/hydrogel nanocomposites with anisotropic structure. Macromol. Chem. Phys. 223, 2100117. doi: 10.1002/macp.202100117

[B14] Gentry M. (2019). Local heat, local food: Integrating vertical hydroponic farming with district heating in Sweden. Energy 174, 191–197. doi: 10.1016/j.energy.2019.02.119

[B15] Hamilton K. (2018). “Struggling with lettuce: Germination,” in Extension Master Gardener Volunteers of Durham County Blog. Available at: https://durhammastergardeners.com/2018/09/06/struggling-with-lettucegermination/.

[B16] Hayashi E. Aoyama N. Still D. W. (2008). Quantitative trait loci associated with lettuce seed germination under different temperature and light environments. Genome 51, 928–947. doi: 10.1139/g08-077 18956026

[B17] Hosseini H. Mozafari V. Roosta H. R. Shirani H. van de Vlasakker P. C. H. Farhangi M. (2021). Nutrient use in vertical farming: Optimal electrical conductivity of nutrient solution for growth of lettuce and basil in hydroponic cultivation. Horticulturae 7, 283. doi: 10.3390/horticulturae7090283

[B18] Huang Y. Yu L. Jiang L. Shi X. Qin H. (2022). 3d printing of hydrogel-based seed planter for in-space seed nursery. Manuf. Lett. 33, 103–108. doi: 10.1016/j.mfglet.2022.07.045

[B19] Jiang X. Huang Y. Cheng Y. Zhang Z. Shi X. Qin H. (2021). Effects of lyophilization on the release profiles of 3d printed delivery systems fabricated with carboxymethyl cellulose hydrogel. Polymers 13, 749. doi: 10.3390/polym13050749 33670898 PMC7957655

[B20] Lee E. Rout P. R. Bae J. (2021). The applicability of anaerobically treated domestic wastewater as a nutrient medium in hydroponic lettuce cultivation: Nitrogen toxicity and health risk assessment. Sci. Total Environ. 780, 146482. doi: 10.1016/j.scitotenv.2021.146482 33770595

[B21] Li J. Wu T. Huang K. Liu Y. Liu M. Wang J. (2021). Effect of LED spectrum on the quality and nitrogen metabolism of lettuce under recycled hydroponics. Front. Plant Sci. 12. doi: 10.3389/fpls.2021.678197 PMC8247776 34220897

[B22] Luo A. (2020). Evaluation of romaine lettuce (lactuca sativa l. Cv. Parris island) production under an elevated carbon dioxide (CO2) gas environment generated from compost materials. [Ph.D. thesis]. Dalhousie University.

[B23] Ma L. Shi Y. Siemianowski O. Yuan B. Egner T. K. Mirnezami S. V.. (2019). Hydrogel-based transparent soils for root phenotyping in *vivo* . Proc. Natl. Acad. Sci. 116, 11063–11068. doi: 10.1073/pnas.1820334116 31088969 PMC6561166

[B24] Magwaza S. T. Magwaza L. S. Odindo A. O. Mditshwa A. (2020). Hydroponic technology as decentralised system for domestic wastewater treatment and vegetable production in urban agriculture: A review. Sci. Total Environ. 698, 134154. doi: 10.1016/j.scitotenv.2019.134154 31505342

[B25] Majid M. Khan J. N. Shah Q. M. A. Masoodi K. Z. Afroza B. Parvaze S. (2021). Evaluation of hydroponic systems for the cultivation of lettuce (lactuca sativa l., var. longifolia) and comparison with protected soil-based cultivation. Agric. Water Manage. 245, 106572. doi: 10.1016/j.agwat.2020.106572

[B26] Millar K. D. L. Kumar P. Correll M. J. Mullen J. L. Hangarter R. P. Edelmann R. E.. (2010). A novel phototropic response to red light is revealed in microgravity. New Phytol. 186, 648–656. doi: 10.1111/j.1469-8137.2010.03211.x 20298479

[B27] Moeinizade S. Hu G. Wang L. (2022). A reinforcement learning approach to resource allocation in genomic selection. Intell. Syst. Appl. 14, 200076. doi: 10.1016/j.iswa.2022.200076

[B28] Moursi Y. S. Thabet S. G. Amro A. Dawood M. F. A. Baenziger P. S. Sallam A. (2020). Detailed genetic analysis for identifying QTLs associated with drought tolerance at seed germination and seedling stages in barley. Plants 9, 1425. doi: 10.3390/plants9111425 33114292 PMC7690857

[B29] Oladosu Y. Rafii M. Y. Arolu F. Chukwu S. C. Salisu M. A. Fagbohun I. K.. (2022). Superabsorbent polymer hydrogels for sustainable agriculture: A review. Horticulturae 8, 605. doi: 10.3390/horticulturae8070605

[B30] Peiro E. Pannico A. Colleoni S. G. Bucchieri L. Rouphael Y. Pascale S. D.. (2020). Air distribution in a fully-closed higher plant growth chamber impacts crop performance of hydroponicallygrown lettuce. Front. Plant Sci. 11. doi: 10.3389/fpls.2020.00537 PMC7237739 32477383

[B31] Saad M. H. M. Hamdan N. M. Sarker M. R. (2021). State of the art of urban smart vertical farming automation system: Advanced topologies, issues and recommendations. Electronics 10, 1422. doi: 10.3390/electronics10121422

[B32] Sapkota S. Sapkota S. Liu Z. (2019). Effects of nutrient composition and lettuce cultivar on crop production in hydroponic culture. Horticulturae 5, 72. doi: 10.3390/horticulturae5040072

[B33] Serrano R. Gaxiola R. (1994). Microbial models and salt stress tolerance in plants. Crit. Rev. Plant Sci. 13, 121–138. doi: 10.1080/07352689409701911

[B34] Shrivastava A. Nayak C. K. Dilip R. Samal S. R. Rout S. Ashfaque S. M. (2023). Automatic robotic system design and development for vertical hydroponic farming using IoT and big data analysis. Mater. Today: Proc. 80, 3546–3553. doi: 10.1016/j.matpr.2021.07.294

[B35] Sihombing P. Zarlis M. Herriyance (2019). “Automatic nutrition detection system (ANDES) for hydroponic monitoring by using micro controller and smartphone android,” in 2019 Fourth International Conference on Informatics and Computing (ICIC) (IEEE). doi: 10.1109/icic47613.2019.8985851

[B36] Tang T.-C. Tham E. Liu X. Yehl K. Rovner A. J. Yuk H.. (2021). Hydrogel-based biocontainment of bacteria for continuous sensing and computation. Nat. Chem. Biol. 17, 724–731. doi: 10.1038/s41589-021-00779-6 33820990 PMC9269716

[B37] Tetreault J. Fogle R. L. Fogarty S. Guerdat T. (2023). Coupled aquaponics: Optimizing hydraulic retention times using a parallel unit process water treatment approach. Front. Hortic. 2. doi: 10.3389/fhort.2023.1140998

[B38] Thapa R. Tabien R. E. Thomson M. J. Septiningsih E. M. (2020). Genome-wide association mapping to identify genetic loci for cold tolerance and cold recovery during germination in rice. Front. Genet. 11. doi: 10.3389/fgene.2020.00022 PMC7047875 32153631

[B39] Velazquez-Gonzalez R. S. Garcia-Garcia A. L. Ventura-Zapata E. Barceinas-Sanchez J. D. O. Sosa-Savedra J. C. (2022). A review on hydroponics and the technologies associated for medium- and small-scale operations. Agriculture 12, 646. doi: 10.3390/agriculture12050646

[B40] Wang L. (2022). The leave-worst-k-out criterion for cross validation. Optim. Lett. 17, 545–560. doi: 10.1007/s11590-022-01894-6

[B41] Yang T. Kim H.-J. (2020). Comparisons of nitrogen and phosphorus mass balance for tomato-, basil-, and lettuce-based aquaponic and hydroponic systems. J. Clean. Prod. 274, 122619. doi: 10.1016/j.jclepro.2020.122619

[B42] Yasin M. Andreasen C. (2016). Effect of reduced oxygen concentration on the germination behavior of vegetable seeds. Hortic. Environ. Biotechnol. 57, 453–461. doi: 10.1007/s13580-016-0170-1

[B43] Zabel P. Zeidler C. Vrakking V. Dorn M. Schubert D. (2020). Biomass production of the EDEN ISS space greenhouse in Antarctica during the 2018 experiment phase. Front. Plant Sci. 11. doi: 10.3389/fpls.2020.00656 PMC7264257 32528506

[B44] Zhang S. Guo Y. Li S. Ke Z. Zhao H. Yang J.. (2022). Investigation on environment monitoring system for a combination of hydroponics and aquaculture in greenhouse. Inf. Process. Agric. 9, 123–134. doi: 10.1016/j.inpa.2021.06.006

